# Magnetic nanoparticles tethered with Zn–DPA for the removal of bacteria from red blood cell suspension

**DOI:** 10.1039/d5ra03701h

**Published:** 2025-09-02

**Authors:** Tochukwu P. Okonkwo, Rajendra P. Gautam, Jacob B. Limburg, Breckin L. Forstrom, Bowen J. Houser, Aaron Rappleyea, Tyler P. Green, Joseph P. Talley, Alexander D. Daum, Stacey J. Smith, Karine Chesnel, William G. Pitt, Roger G. Harrison

**Affiliations:** a Department of Chemistry and Biochemistry, Brigham Young University Provo UT 84602 USA roger_harrison@byu.edu tokonkwo02@gmail.com jacoblimburg2@gmail.com adaum4@gmail.com stacey.smith@byu.edu; b Department of Physics and Astronomy, Brigham Young University Provo UT 84602 USA gautamrp1@gmail.com kchesnel@byu.edu; c Department of Chemical and Biological Engineering, Department of Life Sciences, Brigham Young University Provo UT 84602 USA breckinf@student.byu.edu bowen.houser@gmail.com aaron.rappleyea@byu.edu aawindfall@gmail.com talleyj2@byu.edu pitt@byu.edu

## Abstract

Bacterial infections continue to drive the need for more effective and rapid methods for bacterial analysis. To address this, magnetic nanoparticles (MNPs) have emerged as promising tools, especially when their surfaces are modified with bacteria binders. The bis-zinc–dipicolylamine (Zn–DPA) complex is known for its broad affinity to bacteria. We have synthesized MNPs *via* a thermal decomposition method, encapsulated them in silica, modified their surface with Zn–DPA, and tested their ability to remove bacteria. The MNPs retain their superparamagnetic properties and crystallite structure after being encapsulated. The MNPs coated with silica and Zn–DPA effectively bind and remove both Gram-positive and Gram-negative bacteria from bacterial suspensions in both PBS buffer and red blood cell suspension. The capture efficiency (CE) of bacteria is high, >0.95 for both concentrated (1 × 10^8^ CFU) and dilute (1 × 10^3^ CFU) suspensions of Gram-positive and Gram-negative bacteria in PBS. The bacterial capture efficiency in red blood cell suspension with 50% hematocrit ranges is high (CE > 0.95) for both concentrated and dilute suspensions of *S. aureus* but lower for concentrated (CE = 0.30) and dilute (CE = 0.15) suspensions of *E. coli*. The Zn–DPA coated MNPs have promising binding efficiencies for a broad-spectrum of bacteria within a short period of time, potentially leading to applications in diagnostic devices for both medical and industrial uses.

## Abstract 2

Gram-negative and Gram-positive bacteria capture by functionalized magnetic nanoparticles in buffer and red blood cell suspension.

## Graphical abstract

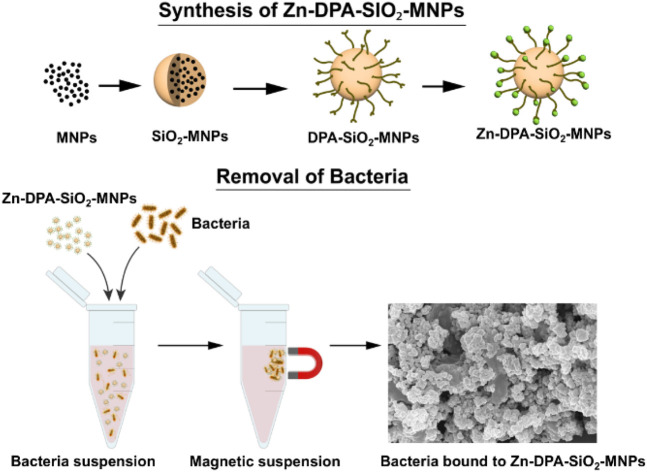

## 1 Introduction

With increasing mortality due to bacterial infections by multidrug resistance bacteria, more effective bacterial analysis has become increasingly important. To meet the need for better bacterial infection treatment by antibiotics, more rapid identification of bacteria from body fluids is needed. Quicker treatment with an appropriate antibiotic is associated with a significantly decreased mortality rate.^1^ The common culturing method to detect bacterial species and antibiotic susceptibility is sensitive and specific,^2,3^ but it takes several days, which prevents its real-time feedback to clinicians.^4,5^ Hence, there is a need for a fast method to detect bacteria in body fluids to reduce the looming threat of inappropriate antibiotic treatment for bacterial infections.

Magnetic nanoparticles (MNPs) have garnered attention in many applications due to their unique properties which include easy preparation, superparamagnetism, large surface area, surface modifications, and promising biocompatibility.^6–8^ Magnetic separation stands out as a favored technique, as it offers such benefits as simplicity, affordability, sensitivity, and the opportunity for additional modifications.^9,10^ To enhance the specificity and sensitivity of MNPs in targeting bacteria, ligands, peptides, proteins, antibodies, and aptamers are used to modify the surface of MNPs. This creates affinity probes designed for bacteria detection and isolation.^11–19^ Also, surface functionalization alters the MNPs surface charge to prevent agglomeration and oxidation and makes them compatible with their intended application. Silica-coatings have been reported to prevent superparamagnetic core aggregation and improve the stability and biocompatibility of MNPs.^20,21^ The stabilizing function of the silica on MNPs occurs by shielding the magnetic dipole interactions of MNPs and by creating a negatively charged surface, resulting in enhancement of coulombic repulsions by the silica-coated MNPs. Additionally, the silica layer has silanol groups that readily react with compounds to enable attachment of compounds to its surface.^22–27^

The zinc–dipicolylamine (Zn–DPA) complex has broad affinity towards nearly all bacterial cell membranes and binds to the anionic phospholipids present on the outer membrane of both Gram-positive and Gram-negative bacteria.^28–30^ Being a small molecule, the Zn–DPA presents a cheaper and more stable alternative to proteins and other more complex molecules. The DPA ligand is known to form stable complexes with metal cations; for example, the Zn^2+^ ion coordinates strongly to DPA.^31^ The bacteria affinity of the Zn–DPA group has been used to optically image bacteria and target the site of bacterial infection.^32–35^ Fluorescent silica nanoparticles (FSiNP) modified with metal–dipicolylamine (M–DPA) groups were synthesized and used for the detection of *S. aureus*.^36^ The result showed that the M–DPA–FSiNP complex efficiently formed aggregates with *S. aureus* and had antibacterial activity. We thought of combining the bacteria binding ability of Zn–DPA with the bacteria removal properties of MNPs. To our knowledge, there has been only one previous example of this where Lee *et al.* modified MNPs with a PEG polymer that contained Zn–DPA and placed the coated MNPs in a magnetic microfluidic device to remove *E. coli*.^28^ The devices worked fairly well; however, MNP build-up reduced the efficiency and the flow of fluids through the device. To facilitate broader applications, there is a need to investigate Zn–DPA–MNPs for binding to a wide variety of bacteria. Also, it would be beneficial to not have a polymer coating on the surface of the MNPs, as polymers have shown binding to proteins.^37^

Herein, we describe in detail the procedure to synthesize monodisperse MNPs, encapsulate them in silica (SiO_2_–MNPs), and attach Zn–DPA to their surface (Zn–DPA–SiO_2_–MNPs). We show that this new material efficiently binds high and low concentrations Gram-negative and Gram-positive bacteria, facilitating the removal of bacteria from phosphate buffer saline solution (PBS) and suspensions of red blood cells in PBS. We found that the superparamagnetic behavior of the MNPs remained the same after encapsulation in silica. We characterized the new material using IR, SEM, TEM, XRD, XPS, DLS, and magnetometry. The ability of the Zn–DPA functionalized MNPs to bind and capture bacteria could potentially be applied in diagnostic devices for broad spectrum collection and rapid identification of bacteria in medical applications and industrial processes.

## 2 Experimental methods

### 2.1 Reagents and materials

The synthesis was carried out using commercially available reagents. Absolute ethanol, hexane, toluene, ammonium hydroxide, methanol, and acetone were used as received. Benzyl ether (99%), 1,2-hexadecanediol (97%), tetramethyl orthosilicate (TMOS), iron(iii) acetylacetonate (Fe(acac)_3_), and di-(2-picolyl)amine were purchased from Sigma-Aldrich Chemical Co. 3-Aminopropyltrimethoxysilane (APTMS) was purchased from Tokyo Chemical Industries, Japan. Heptanoic acid (90%) was purchased from Matheson Coleman and Bell Company. Butylamine (98%) was purchased from EM Industries Inc. Bis-DPA–SVA was synthesized by modifying an existing procedure (see SI^28^). Phosphate buffered saline (PBS) 1× without calcium and magnesium, pH 7.4 ± 0.1 was purchased from Corning.

The instruments used for characterization included: Thermo Scientific Nicolet iS5 FTIR spectrometer, Thermo Scientific Verios G4 UC SEM (Thermo Scientific, Waltham, MA, USA), Tecnai TF-20 TEM (FEI, Hillsboro, OR, USA), Q150T-ES thin-film coater (QUORUM, Sacramento, CA, USA), Q150T-ES thin-film coater (QUORUM, Sacramento, CA, USA), K-Alpha X-ray Photoelectron Spectrometer (Thermo Scientific, Waltham, MA, USA), Panalytical X'Pert Pro MPD X-ray diffractometer (Malvern Panalytical, Westborough, MA, USA), Zetasizer Nano Zs Dynamic light scattering instrument (Malvern Instruments, Worcestershire, MA, USA), vibrating sample magnetometry (VSM) (Quantum Design, San Diego, CA, USA), Olis 8453 Diode UV/vis spectrophotometer (Olis, Athens, GA, USA) and Cary 60 UV/vis spectrometer (Agilent, Santa Clara, CA, USA).

### 2.2 Synthesis of MNPs

Fe_3_O_4_ magnetic nanoparticles were synthesized *via* a thermal decomposition method by modifying existing procedures.^38^ Fe(acac)_3_ (2.0 mmol), 1,2-hexadecanediol (10 mmol), benzyl ether (20 mL), heptanoic acid (2 mmol), and butylamine (2.0 mmol) were mixed and stirred magnetically. The solution was heated to 200 °C for 2 h under a nitrogen atmosphere and then heated to reflux at 300 °C for 1 h. The black colored mixture was allowed to cool to room temperature, and the MNPs were separated with a strong magnet, washed with ethanol and hexanes, centrifuged between washes, dried, collected and weighed (mass 86.5 mg, % yield 56%).

### 2.3 Silica encapsulated MNPs

To encapsulate the MNPs in silica, we modified a literature procedure.^39^ MNPs (4.6 mg) were dispersed in 1 mL of toluene and sonicated for 30 min in a bath sonicator. The MNP solution was added to a mixture of isopropanol and propanol (10 mL each), 2.5 mL deionized water, and 0.50 mL NH_4_OH (28–30%) and stirred magnetically for 10 min at 30 °C. Tetramethyl orthosilicate (TMOS, 0.80 mL) was added dropwise to the reaction mixture and stirred for 30 min. Solid coated MNP material was collected at the bottom of the flask using a magnet, washed with a 3 : 1 ethanol/water solution, and dried in an oven at 120 °C for 1 h. The material mass was 14.2 mg (4.31% yield).

### 2.4 Synthesis of Zn–DPA–SiO_2_–MNPs

The functionalization of the SiO_2_–MNPs with Zn–DPA was carried out by adopting a procedure to add DPA–SVA to coated silica beads, followed by Zn incorporation.^40^ First, amino groups were attached to SiO_2_–MNPs by dispersing 60 mg of SiO_2_–MNPs in 3 mL of anhydrous toluene and adding 221 μL of APTMS to the mixture. The mixture was stirred at refluxed (110 °C) for 24 h. The obtained particles (NH_2_–SiO_2_–MNPs) were separated using a magnet and then sequentially washed with toluene, methanol, and acetone. The isolated NH_2_–SiO_2_–MNPs yielded 56.4 mg (94.0% yield).

To attach bis-DPA, NH_2_–SiO_2_–MNPs (60 mg) were added to bis-DPA–SVA (75 mg, 1.1 mmol), EDC (25 mg, 1.3 mmol), HOBt·H_2_O (19.8 mg, 1.3 mmol), and DIEA (46 mg, 3.57 mmol) in dry DMF (2.0 mL) and stirred at room temperature for 3 days. The DPA–SiO_2_–MNPs were separated with a magnet and sequentially washed with toluene, methanol, and acetone. The DPA–SiO_2_–MNPs obtained after washing had a mass of 50 mg, (47.5% yield). Then, DPA–SiO_2_–MNPs (28.5 mg) were dispersed in 2.0 mL of a water/MeOH (1 : 1) solution, and a solution of Zn(NO_3_)_2_·6H_2_O (0.15 g, dissolved in 2.0 mL water/MeOH, 1 : 1) was added dropwise. The reaction was stirred at room temperature for 72 h and Zn–DPA–SiO_2_–MNPs were separated with a magnet and sequentially washed with water, ethanol, and acetone. The dried Zn–DPA–SiO_2_–MNPs yielded 24.4 mg (85.6% yield).

### 2.5 Characterization of materials

FTIR KBr pellets were prepared with dry MNPs, SiO_2_–MNPs, NH_2_–SiO_2_–MNPs and DPA–SiO_2_–MNPs in a 5 : 95 mg ratio of sample to KBr and analyzed using a FTIR spectrometer.

The SEM and TEM samples were prepared by making 0.20 mg mL^−1^ of MNPs, SiO_2_–MNPs and Zn–DPA–SiO_2_–MNPs samples dispersed in ethanol for SEM and toluene for TEM. For SEM visualization, 10 μL of MNPs, SiO_2_–MNPs and Zn–DPA–SiO_2_–MNPs suspensions were transferred to a silicon wafer and dried for 24 h in a desiccator. For SEM visualization of some samples such as SiO_2_–MNPs, NH_2_–SiO_2_–MNPs, Zn–DPA–SiO_2_–MNPs and Zn–DPA–SiO_2_–MNPs bound to bacteria, Au/Pd was sputtered on the samples using a Q150T S sputter coater instrument for 60 s to form a 10.0 nm layer of Au/Pd. They were imaged with the SEM instrument at a voltage of 5–10 kV.

For TEM imaging, 5.0 μL of suspensions were transferred to an ultra-thin carbon film on a holey/lacey film, 400 mesh, Cu grid, and dried in a desiccator for 24 h. The images obtained from both SEM and TEM were processed with ImageJ software (NIH, Bethesda, MD, USA) to obtain the diameters of the particles.

X-ray photoelectron spectroscopy (XPS) was carried out by attaching double-sided tape to a silicon wafer. Dry MNPs, SiO_2_–MNPs and Zn–DPA–SiO_2_–MNPs were placed on the upper side of the tape and a XPS spectra was obtained. The data was processed using CaxaXPS software (Casa Software Ltd, Teignmouth, Devon, UK).

X-ray diffraction data were collected using an X'Celerator detector and a Cu X-ray source with a Ge monochromator selecting the Kα1 wavelength (*λ* = 1.5406 Å). Fixed divergence and antiscatter slits (0.25°), soller slits (0.04 rad), and a mask (10 mm) were used to condition the beam. The PHD lower and upper levels of the detector were adjusted to 55% and 80%, respectively, to avoid Fe fluorescence. The MNPs and silica-coated were loaded into a zero-background holder and scanned between 14° and 124° 2*θ* with a step size of 0.0084° per step and a counting time of 350 s per step. Data for a NIST LaB_6_ line position and line shape standard (660b) were collected over the same range of 2*θ* using the same instrument configuration. The data were matched to Fe_3_O_4_ magnetite (PDF #04-015-9120) and cristobalite SiO_2_ patterns (PDF #04-025-9060) in the ICDD WebPDF-4+ database. Both the LaB_6_ standard reference material and the Fe_3_O_4_ were profile fit with the Highscore Plus software using Pearson VII profile functions and polynomial backgrounds. The Gaussian and Lorentzian coefficients from the LaB_6_ profile fit were used to estimate and subtract the instrument contribution to the peak width in the Fe_3_O_4_ sample. A linear Williamson–Hall plot was used to estimate the size and strain contributions to the peak broadening in the MNPs and SiO_2_–MNPs samples.

DLS measurements were done by dispersing 0.20 mg mL^−1^ of the MNPs, SiO_2_–MNPs and Zn–DPA–SiO_2_–MNPs in ethanol at room temperature to obtain the zeta potentials of the MNPs samples.

### 2.6 Magnetometry

The magnetometry data was acquired using vibrating sample magnetometry (VSM) with a Physical Properties Measurement System (Quantum Design, San Diego, USA), that includes a superconducting magnet capable of generating magnetic fields up to 9 T and a cryostat that allows temperature variations from 10–400 K. For zero field cooling (ZFC) measurements, the sample was cooled to 10 K without an applied magnetic field. Subsequently, a magnetic field of 100 Oe was applied, and magnetization was recorded as the sample was heated to 400 K. For field cooling (FC) measurements a 100 Oe magnetic field was applied while cooling the sample down to 10 K, and magnetization data was collected as the sample was warming up to 400 K. The magnetization loops were recorded by sweeping the magnetic field typically between −7000 Oe and +7000 Oe at fixed temperature.

### 2.7 Beads per volume

To determine the concentration of nanoparticles used to bind bacteria, the radius (140 nm) of the beads was used to calculate the volume (1.15 × 10^−20^ m^3^) of each bead. Assuming that the silicate has a density of 2.2 g cm^−3^, a concentration of 2.0 mg beads per mL of suspension has a number density of 7.9 × 10^10^ beads per mL of suspension.

### 2.8 pH stability

A 10 mg sample of Zn–DPA–SiO_2_–MNPs was dispersed in 2.0 mL of PBS solutions at different pH values (2, 5, 7, 9, and 12). The samples were sonicated for 5 minutes, allowed to sit for 1 h and the Zn–DPA–SiO_2_–MNPs were collected with a magnet. The supernatants of pH 5, 7, and 9 were clear, while those of pH 2 and 12 were cloudy. The supernatants were analyzed by UV/vis spectroscopy and the solid MNPs by XPS.

### 2.9 Bacterial preparation

Five strains of bacteria were studied: *Staphylococcus aureus* (*S. aureus*, strain ATCC #12600), *Staphylococcus epidermidis* (*S. epidermidis*, strain RP62A), *Pseudomonas aeruginosa* (*P. aeruginosa*, strain PAO1), *Escherichia coli* (*E. coli*) DH5α and *E. coli* ATCC #25922. In preliminary experiments, a correlation for each species was done between optical density at 600 nm (OD_600_) and bacterial concentration in colony forming units (CFU) per mL, as measured by plate counting.

All strains were streaked from frozen culture onto an appropriate plate, grown for 4 h at 37 °C, and a single colony was used to inoculate 20 mL of appropriate growth media. After overnight culture, a 200 μL aliquot was used to inoculate 20 mL of fresh growth media in a shaker flask. The flask was shaken on an orbital shaker at 150 rpm and incubated at 37 °C until the culture was in the logarithmic phase of growth. See Table S1 for growth times used to attain log-phase phenotype.

After the appropriate growth time, the suspension was harvested and centrifuged 10 minutes at 488*g* to form a pellet. The pellet was washed twice by resuspending the bacteria in about 2–3 mL of PBS, followed by vortexing and then centrifuging for 10 minutes at 488*g*. After the final resuspension of the pellet, the bacterial suspension was diluted with PBS to give an optical density (OD) reading between 0.5 and 1.2, as measured on a Cary 60 UV/vis spectrometer at 600 nm (OD_600_). An estimated 1 × 10^8^ CFU mL^−1^ was prepared for final use.

### 2.10 Bacteria capture

Bacterial suspensions of 1.3 × 10^8^ CFU mL^−1^ as estimated by OD_600_ were used. One milliliter of suspension was added to five 1.5 mL microcentrifuge tubes. Dispersions of 2.0 mg mL^−1^ of MNPs in PBS were prepared, sonicated for 15 minutes, and vortexed before being added to the bacteria suspensions. Aliquots of 0.300 mL of the 2.0 mg per mL MNP dispersions were added to four of the 1.00 mL of bacteria suspension tubes and 0.300 mL of PBS was added to the control tube. This diluted the bacterial suspensions to 1.3 × 10^8^ CFU mL^−1^. The solids in the samples were kept in suspension by vortexing followed by mounting on a platform that slowly rotated the microcentrifuge tubes end-over-end at 30 rpm. After 15 minutes, each tube was placed in a rack with a single neodymium magnet positioned very close (∼1 mm) to the outer surface of the tube for 5 min. While the tube was still in the magnetic holder, 1.0 mL of the supernatant was carefully removed by pipette without disturbing the samples collected on the side of the tube. The turbidity of the pipetted liquid (OD_600_) was measured using a UV/vis spectrometer.

The turbidity measurement of the control tube represented the expected turbidity of the bacteria in the other tubes before they were exposed to MNPs. The turbidity in the other four tubes indicated the concentration of bacteria not captured. Capture efficiency (CE) or the fraction of bacteria removed from suspension was calculated using the following equation:1
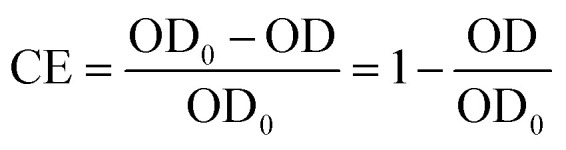
where OD_0_ is the optical density of the control and OD is that of the sample suspension after exposure to MNPs.

### 2.11 Bacteria capture in red blood cell solutions

Whole blood was collected from volunteers into EDTA tubes under an approved protocol (IRB #X2021-135, Brigham Young University, 2024). 6 mL of blood was centrifuged for 5 min after which the plasma and buffy layers were removed. An equivalent volume of PBS was carefully added to the tube and the red blood cells (RBCs) were resuspended by gently inverting the tube by hand. The tube was centrifuged and the RBCs isolated again. This washing step was repeated two more times to remove residual plasma. For high bacteria concentration experiments, RBCs were diluted with PBS to a solution of 50% hematocrit and a PBS solution of bacteria (either *S. aureus* or *E. coli* ATCC #25922) was added to the RBC solution to give a bacteria concentration of 1.3 × 10^8^ CFU mL^−1^. Then, 1.0 mL of RBC/bacteria suspension was added to 5 separate 1.5 mL microcentrifuge tubes. After which 0.30 mL of Zn–DPA–SiO_2_–MNPs suspensions at concentrations of 2.0, 4.0, and 6.0 mg mL^−1^ were added to individual RBC/bacteria suspensions to give a final MNPs concentrations 0.46, 0.92, 1.38 mg mL^−1^. For controls, 0.30 mL of PBS were added to two individual RBC/bacteria suspensions. The bacteria concentration in the suspensions following the addition of the MNP suspension or equivalent volume of PBS was 1.0 × 10^8^ CFU mL^−1^. Next, the tubes were slowly rotated for 15 min, then placed on a magnet for 15 min. While the tube was still on the magnetic holder, supernatant was removed and diluted by standard serial dilution to a target concentration of 1000 CFU mL^−1^. An aliquot of 100 μL of the final dilution was plated onto a nutrient agar plate for each of the 5 tubes. After incubation at 37 °C for 20–28 h, colonies growing on the plates were counted and capture efficiency was calculated using plate counts.

For low bacteria concentration experiments, RBCs were added to PBS to make a 50% hematocrit solution and 6.0 mL of this solution was added to 4.8 mL of 2500 CFU per mL bacteria and gently mixed by inverting the solution by hand. Then 0.90 mL of this RBC/bacteria solution were added to five 1.5 mL tubes and 0.10 mL of 10 mg mL^−1^, 1.0 mg mL^−1^, and 0.10 mg per mL MNP suspensions was added to three of the tubes and PBS to two of them. The final volume was 1.0 mL with a bacteria concentration of 1000 CFU mL^−1^. These were slowly rotated for 15 minutes and were placed on the magnet for another 15 minutes. Following, 0.10 mL of each test were plated and allowed to incubate for 20–28 h at 37 °C, following the same counting method as previously mentioned. The binding studies were done in triplicate.

## 3 Results and discussion

### 3.1 Synthesis of Zn–DPA–SiO_2_–MNPs

**Fig. 1:**
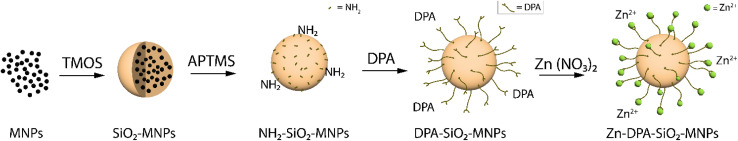
Synthetic scheme for Zn–DPA–SiO_2_–MNPs. The MNPs were encapsulated with silica, and aminated, afterwards DPA was added to the aminated beads followed by Zn^2+^ coordination.

Monodispersed 8.4 nm MNPs were synthesized by a thermal decomposition method. Fe(acac)_3_ in the presence of 2-hexadecanediol, benzyl ether, heptanoic acid, and butylamine was heated to 300 °C.^38,41–43^ The MNPs were encapsulated in silica by adding TMOS to a solution containing water, alcohol, NH_4_OH and MNPs in toluene (SiO_2_–MNPs, Fig. 1). We found that TMOS, instead of the commonly used triethyl orthosilicate (TEOS), and a combination of isopropanol and propanol were necessary to form silica beads with diameters of around 300 nm. Beads of around 300 nm were desired to facilitate the binding of several beads around a single bacterium, which is on the order of 1 to 2 μm in size. Larger silica beads, around one micron formed when TEOS and only isopropanol were used. Amino groups were added to the surface of the silica beads to provide attachment sites for DPA. The amino groups were bonded to the surface by adding APTMS to a dispersion of SiO_2_–MNPs in anhydrous toluene. The addition of DPA and formation of DPA–SiO_2_–MNPs was carried out by adding NH_2_–SiO_2_–MNPs to a DMF solution of EDC, NHS, DIEA, and bis-DPA–SVA at room temperature. Finally, Zn^2+^ was coordinated to DPA by adding Zn(NO_3_)_2_·6H_2_O to DPA–SiO_2_–MNPs in a methanol/water solution at room temperature.

### 3.2 Characterization of materials

**Fig. 2:**
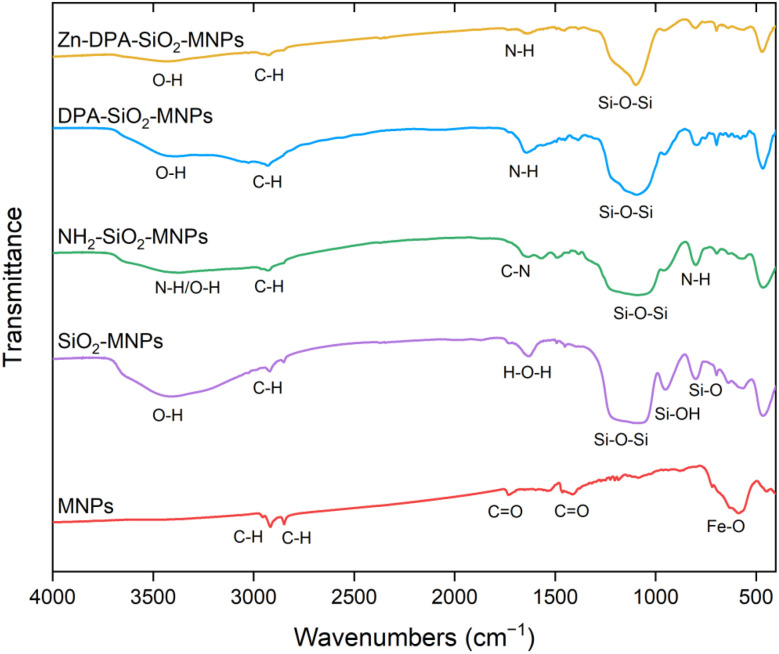
FTIR spectrum of the MNPs, SiO_2_–MNPs, NH_2_–SiO_2_–MNPs, DPA–SiO_2_–MNPs, and Zn–DPA–SiO_2_–MNPs.

The MNP materials were characterized to confirm the successful completion of each synthetic step, to estimate the size and morphology of the MNPs, and to study their magnetic properties both before and after modifications. FTIR analysis of the MNPs showed the presence of an Fe–O stretch at ∼586 cm^−1^ (Fig. 2). It also showed peaks at ∼2916 and 2848 cm^−1^ indicating the presence of C–H groups, most likely from heptanoic acid molecules on the surface of the MNPs. The encapsulation of the MNPs with silica was confirmed by the presence of a strong peak for the Si–O–Si symmetric stretch at ∼1097 cm^−1^ and the Si–OH stretching shoulder at around 951 cm^−1^. The SiO_2_–MNPs spectrum also shows the presence of the –OH peak at ∼3406 cm^−1^ indicating the presence –OH group on the silica surface.^23,44–46^ The NH_2_–SiO_2_–MNPs spectrum showed the presence of a characteristic C–N vibration peak at 1570 cm^−1^ giving evidence of a successful modification of the SiO_2_–MNPs with NH_2_ groups from APTMS. It also had a peak at 2928 cm^−1^, which is assigned to C–H stretching vibrations of propyl groups from APTMS.^47,48^ The Si–O–Si symmetric peaks also appeared in the spectrum of NH_2_–SiO_2_–MNPs. The DPA–SiO_2_–MNPs spectrum showed the presence of a characteristic peak for NH at 1641 cm^−1^ attributed to N–H bending showing a successful modification of the NH_2_–SiO_2_–MNPs with DPA. The peak at 3402 cm^−1^ and 2930 cm^−1^ are assigned to the –OH and C–H stretching vibration of DPA. The Zn–DPA–SiO_2_–MNPs spectrum showed the presence of similar peaks to those from DPA–SiO_2_–MNPs.

**Fig. 3:**
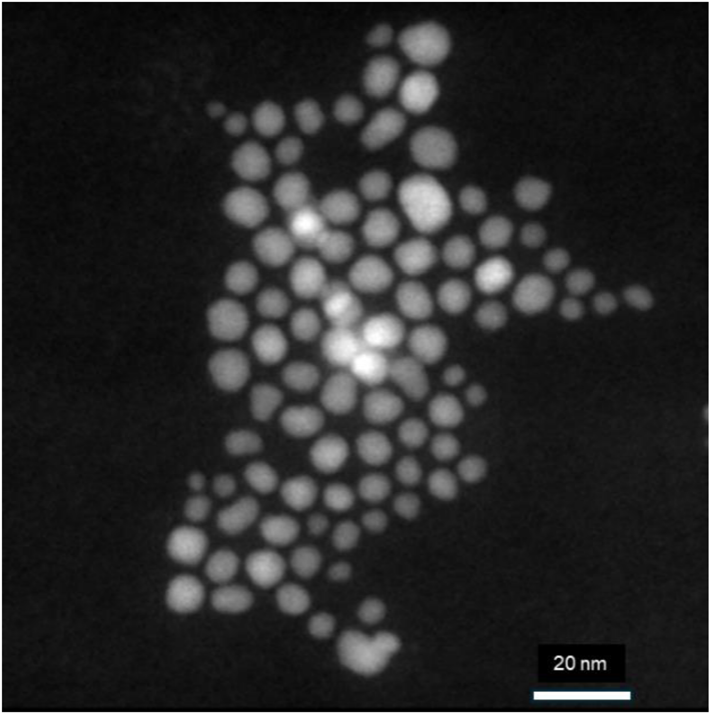
TEM image of MNPs showing monodispersed 8.4 nm MNPs.

**Fig. 4:**
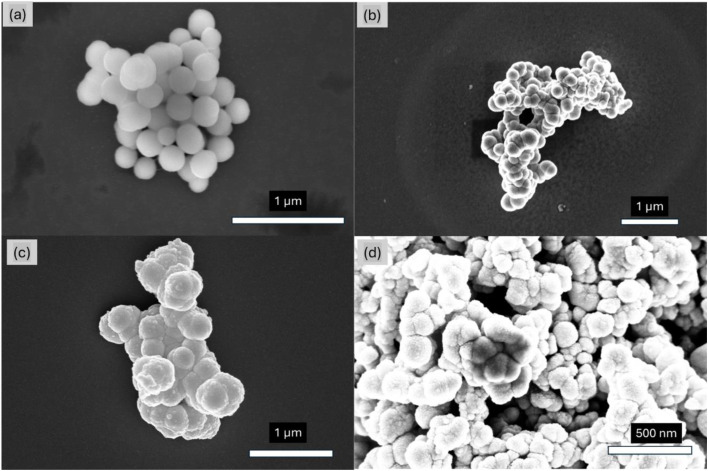
SEM images of (a) SiO_2_–MNPs (b) SiO_2_–MNPs sputtered with Au/Pd (c) NH_2_–SiO_2_–MNPs sputtered with Au/Pd and (d) Zn–DPA–SiO_2_–MNPs sputtered with Au/Pd of 20 nm thickness.

The morphology and size of the MNP materials were analyzed using SEM and TEM. Fig. 3 shows a TEM micrograph of the monodispersed MNPs and shows their 8.4 ±1.3 nm (*n* = 100) average diameter. Fig. 4 and b shows SEM images of SiO_2_–MNPs. The silica coated MNPs are well defined spheres with average diameters of 280 ± 10 nm (*n* = 67). In some images Au/Pd was sputtered on the surface of the spheres to reduce charging while collecting the SEM images. This roughens their surface and masks their spherical structure. Fig. 4c shows NH_2_–SiO_2_–MNPs and Fig. 4d shows Zn–DPA–SiO_2_–MNPs. The SEM images of the NH_2_–SiO_2_–MNPs and Zn–DPA–SiO_2_–MNPs showed the spheres were still intact and maintained their size.

The elemental compositions of the MNPs, SiO_2_–MNPs, NH_2_–SiO_2_–MNPs, DPA–SiO_2_–MNPs and Zn–DPA–SiO_2_–MNPs were determined using XPS (Table 1). The XPS spectra of the MNPs showed peaks for Fe 2p, Fe 2s, Fe 3s, Fe 3p, and O 1s (Fig. S2) with atomic percent of Fe equal to 30.9% and O equal to 47.2%. There was also a peak for C 1s (21.9% C), attributed to the carbon on the surface of the MNPs from the heptanoic acid used as surfactant during synthesis. The SiO_2_–MNPs showed the presence Si 2p (29.6% Si) and a strong signal from O 1s (63.4% O) but no signal from Fe 2p and only a smaller signal from carbon (6.98%) (Fig. S3). The Si signal correlates to the added Si from SiO_2_ and the decrease in Fe and C signals reveal that the MNPs are embedded in the SiO_2_. These beads were isolated with a magnet, so there are MNPs in their interior.

**Table 1:** Elemental composition of MNPs, SiO_2_–MNPs, NH_2_–SiO_2_–MNPs, DPA–SiO_2_–MNPs and Zn–DPA–SiO_2_–MNPs. Values are atom percents found by XPS analysis

Sample	Fe	O	C	Si	N	Zn
MNPs	30.9	47.2	21.9			
SiO_2_–MNPs	0.01	63.4	6.89	29.6		
NH_2_–SiO_2_–MNPs	0.04	50.1	23.1	14.0	12.6	
DPA–SiO_2_–MNPs	0.00	53.5	27.6	10.4	8.44	
Zn–DPA–SiO_2_–MNPs	0.00	56.1	26.3	8.81	2.85	5.84

The XPS spectra after APTMS addition to SiO_2_–MNPs showed a signal from N 1s (12.6%), smaller signals from Si 2p (14.0% Si) and O 1s (50.1%) and a larger signal from C 1s (23.1% C) (Fig. S4). The difference in the signals is explained by the presence of O, Si, C and N, with the Si and O coming from SiO_2_ and the C and N coming from the added propylamine. The XPS spectra after DPA was added to NH_2_–SiO_2_–MNPs showed stronger signals from C 1s (27.6%) and O 1s (53.5%) but weaker signals from Si 2p (10.4%) and N 1s (8.44%) (Fig. S5). The difference in the signals is attributed to the addition of DPA. As it covers the SiO_2_ surface it presents more carbon and oxygen atoms that mask the underlying silicon atoms. The spectra of the Zn–DPA–SiO_2_–MNPs showed peaks for N 1s, C 1s, Si 2p, and O 1s (2.85%, 26.3%, 8.81% and 56.1%) and a strong signal for Zn 2p (5.84%) (Fig. S6), confirming the successful coordination of zinc to DPA.

**Fig. 5:**
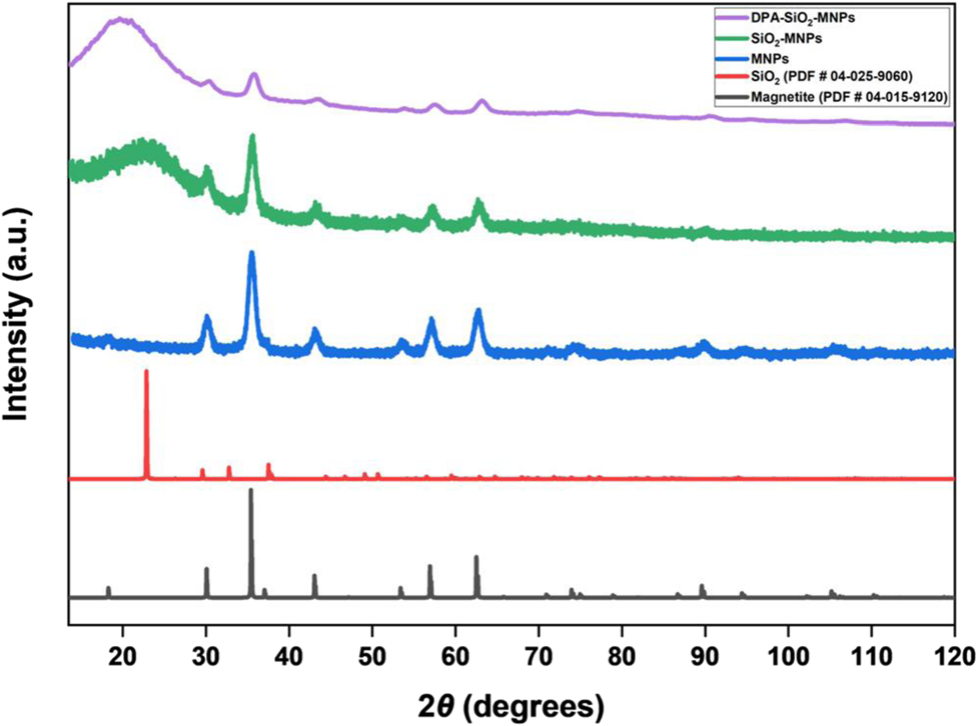
XRD pattern of the MNPs, SiO_2_–MNPs and DPA–SiO_2_–MNPs compared with magnetite (PDF #04-015-9120) and cristobalite silica (PDF #04-025-9060) reference patterns from the ICDD database.

The magnetite structure of the MNPs was confirmed by XRD. Fig. 5 shows the XRD patterns of the MNP materials compared with a magnetite reference pattern (PDF #04-015-9120) from the ICDD WebPDF-4+ database. The MNP, SiO_2_–MNP, and DPA–SiO_2_–MNP materials each display all the peaks anticipated for the magnetite cubic spinel crystal structure including the (111) at 18°, (220) at 30°, (311) at 35°, (400) at 43°, (422) at 53°, (511) at 57°, (440) at 63°, (533) at 74°, and (731) at 90° 2*θ*. Thus, the magnetite crystal structure of the MNPs remains intact during encapsulation in silica and after the addition of DPA to the silica surface. As the MNPs are encapsulated and surface modifications are done, the magnetite signals decrease in intensity.

The size of the magnetite crystals is also not affected by silica encapsulation. Using the XRD data, the average size of the magnetite crystallites was calculated to be 8.3 nm (±1.0 nm) with a strain of 0.10% (±0.01%) before encapsulation and 8.0 nm (±1.4 nm) after encapsulation. These crystallite sizes agree with the MNP size measurements made from TEM images. This suggests that each magnetite crystallite is a single crystal both before and after encapsulation and the size and crystallinity of the magnetite remains the same.

Encapsulation in silica results in broad peaks centered at around 25° for SiO_2_–MNP and 21° for DPA–SiO_2_–MNP. The broad peaks align reasonably well with the most intense peak in the cristobalite silica reference pattern at 22.8° (PDF #04-025-9060). The breadth of the peak indicates that the silica has low crystallinity.^49^

The surface charge on the MNP materials will change as different groups are added to their surface. The zeta potential (*ζ*) was used to study the change in surface charge. The MNPs exhibited a slightly positive surface charge of 6.35 ± 0.5 mV due to the acidic nature of iron(iii) oxide.^50^ Following encapsulation of MNPs with silica, the SiO_2_–MNP's surface charge was −11.7 ± 0.4 mV. The slightly negative value is attributed to hydroxyl groups on the surface of the silica being unprotonated.^51^ Once amine groups are added to the surface, the charge becomes positive, 21.1 ± 0.2 mV. The amine groups will be protonated at the neutral pH of the measurement to give a positively charged surface.^52^ After functionalization with DPA, the positive charge increased to 32.4 ± 2.0 mV, due to the added number of protonated nitrogen's from DPA. After coordinating Zn^2+^ to the DPA–SiO_2_–MNPs, the positive charge again increased to 39.8 ± 0.9 mV, attributed to addition of Zn^2+^ cations.

### 3.3 Magnetic properties of MNPs

**Fig. 6:**
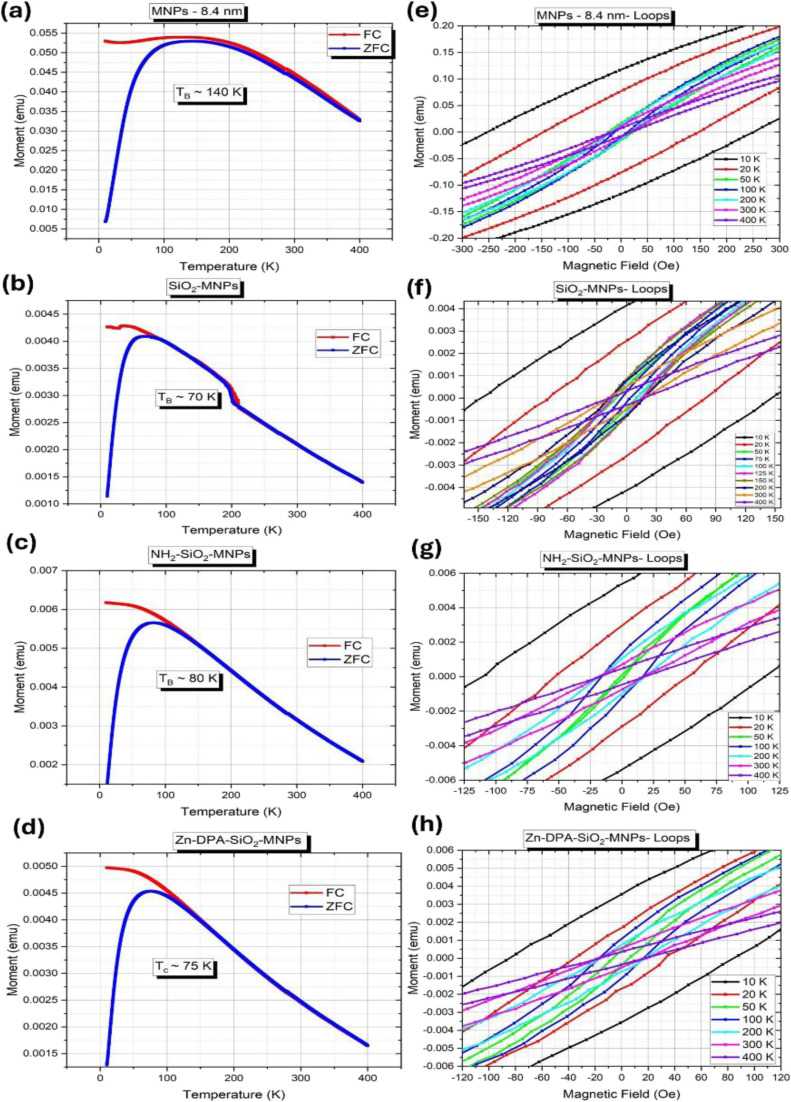
Magnetometry data collected on the MNPs, SiO_2_–MNPs, NH_2_–SiO_2_–MNPs and Zn–DPA–SiO_2_–MNPs. Field cooling (FC/ZFC) curves measured in the range of 20 K to 400 K, (a) MNPs (b) SiO_2_–MNPs (c) NH_2_–SiO_2_–MNPs (d) Zn–DPA–SiO_2_–MNPs. Magnetization loops measured at selected temperatures from 10 K to 400 K, here showing a close-up view in the [−500 Oe, +500 Oe] range on (e) MNPs (f) SiO_2_–MNPs (g) NH_2_–SiO_2_–MNPs (h) Zn–DPA–SiO_2_–MNPs.

Magnetometry measurements were carried on the MNPs to see what effect the silica encapsulation has on their magnetic properties. Fig. 6 shows magnetometry data collected at the various stages of the MNPs functionalization: bare MNPs, MNPs encapsulated in silica beads (SiO_2_–MNPs), aminated beads (NH_2_–SiO_2_–MNPs), and beads coated with Zn–DPA (Zn–DPA–SiO_2_–MNPs). The field cooling (FC) and zero field cooling (ZFC) curves for the bare MNPs (Fig. 6a), indicate that the MNPs are superparamagnetic above a blocking temperature *T*_B_ ∼ 140 K. The extended peak width is likely due to the distribution of particle sizes around 8.4 ± 1.3 nm and indicates strong interparticle magnetic couplings. Once the MNPs are embedded in the silica beads (SiO_2_–MNPs), the shape of the FC–ZFC curves (Fig. 6b) is significantly altered: the curves become somewhat narrower and the peak position is shifted to a lower temperature, around 70 K, which is closer to the expected value *T*_B_ ∼ 70 K for ∼8 nm isolated (non-interacting) MNPs.^53^ The peak narrowing suggests that interparticle magnetic interactions are significantly reduced once the MNPs are dispersed in the silica beads. After the amination (Fig. 6c) and Zn–DPA coating steps (Fig. 6d), the shapes of the FC–ZFC curves remain practically unchanged compared to the beads, with estimated *T*_B_ ∼ 80 K and 75 K, respectively. The steady measured blocking temperature, remaining in the 70–80 K range for panel (b), (c) and (d), confirms that the chemical coating at the surface of the beads does not affect the magnetic behavior of the MNPs once they are embedded in the silica beads. Indeed, given that the bead size is around 280 nm, what happens at their surface is nearly invisible to the 8 nm MNPs dispersed in the silica matrix, especially as none of the coating elements (NH_2_, DPA, Zn) are magnetic. The magnified magnetization loops in panels (e–h) show a consistent trend. At low temperature below *T*_B_, the MNPs, then in a magnetically blocked state, exhibit some significant magnetic hysteresis: 250 Oe at 10 K for the interacting bare MNPs, down to 150 Oe once embedded in the silica beads and around 100 Oe once coated. However, when above *T*_B_, and in particular at 300 K, the hysteresis is nearly gone (less than 20 Oe) in all cases, thus confirming that the MNPs, both in their bare or coated form, are practically superparamagnetic at 300 K.

### 3.4 pH stability of Zn–DPA–SiO_2_–MNPs

To insure compound stability, Zn–DPA–SiO_2_–MNPs was studied in PBS solutions at different pH values (2, 5, 7, 9 and 12) using UV/vis and XPS. Samples of Zn–DPA–SiO_2_–MNPs were dispersed in PBS at different pH values for 1 hour. After the Zn–DPA–SiO_2_–MNPs was collected with a magnet it was analyzed by XPS. The XPS data of the Zn–DPA–SiO_2_–MNPs gave the following Zn atom percents: pH 2, 2.00%; pH 5, 4.64%; pH 7, 4.85%; pH 9, 4.60%; pH 12, 5.28%. The Zn was still present at all pH values, but decreased significantly at pH = 2. The Zn–DPA–SiO_2_–MNPs supernatant from pH values of 5, 7 and 9 were clear, while solution from pH values of 2 and 12 were cloudy. When the supernatant was analyzed using UV/vis, the solutions from 2 and 12 pH values showed absorption over the entire UV-vis region. These results suggest that Zn–DPA–SiO_2_–MNPs are stable at pH values from 5 to 9 for the duration of a bacteria binding experiment.

### 3.5 Bacteria capture by Zn–DPA–SiO_2_–MNPs

**Fig. 7:**
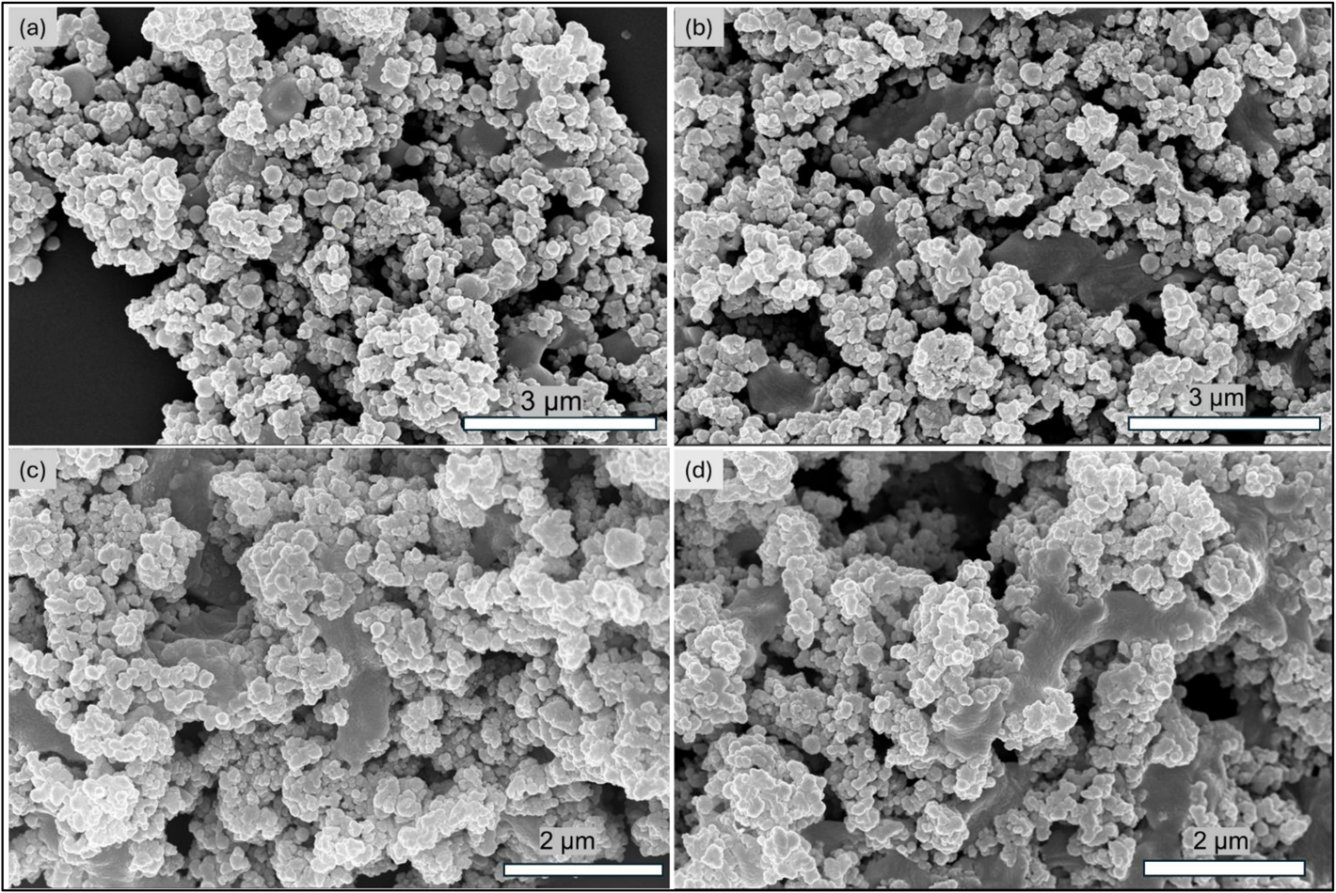
SEM images of the Zn–DPA–SiO_2_–MNPs bound to bacteria (a) *S. aureus* (b) *E. coli* strain DH5α (c) *P. aeruginosa* and (d) *E. coli* strain 25922.

Bacteria were exposed to Zn–DPA–SiO_2_–MNPs aided by vigorous vortexing for a couple of minutes to enable surface to surface interaction of the Zn–DPA–SiO_2_–MNPs with the bacteria. Afterwards, the bacteria bound to Zn–DPA–SiO_2_–MNPs were removed from the solution by a strong magnet. As seen in Fig. 7, a variety of bacteria are bound by Zn–DPA–SiO_2_–MNPs. On application of a strong magnet, the Zn–DPA–SiO_2_–MNPs possess enough force from the magnetic field to quickly pull the bacteria through the solution to the side of the microcentrifuge tube. This process leaves in solution bacteria that did not bind to the Zn–DPA–SiO_2_–MNPs, whose concentration was measured spectroscopically. The capture efficiency of the Zn–DPA–SiO_2_–MNPs of both Gram-negative and Gram-positive bacteria were assessed by measuring the turbidity of the bacteria solution (see eqn (1)).^54,55^

**Fig. 8:**
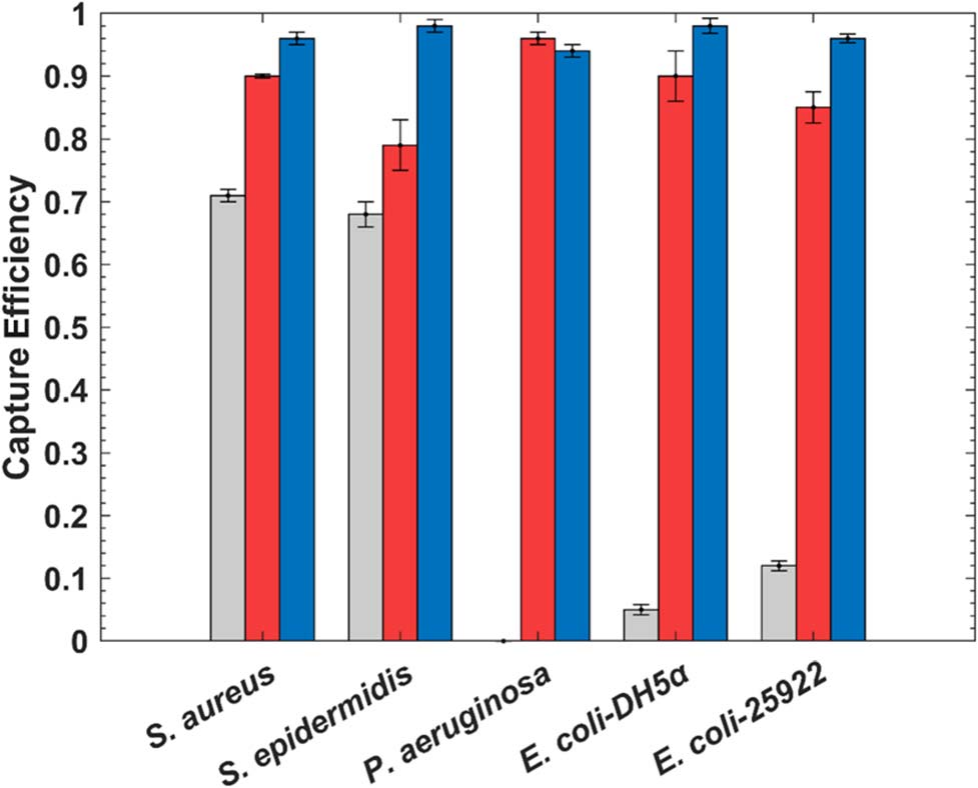
Capture efficiency for bacteria in PBS with SiO_2_–MNPs (grey 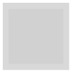), DPA–SiO_2_–MNPs (red 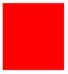), and Zn–DPA–SiO_2_–MNPs (blue 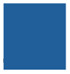). 0.46 mg per mL MNPs dispersions were used with 1.3 × 10^8^ CFU bacteria in PBS. Data are means with the error bars representing the standard deviation (*n* = 3).

The bacteria capture efficiency for Zn–DPA–SiO_2_–MNPs was high for all bacteria studied (>0.94) (Fig. 8). DPA–SiO_2_–MNPs was also tested and its capture efficiencies were lower (0.79–0.96) than those for Zn–DPA–SiO_2_–MNPs except for *P. aeruginosa* which was 0.96. The underivatized MNPs did not disperse in water due to their non-polar surface groups and were thus not efficient in binding bacteria. Surprisingly though, SiO_2_–MNPs showed some ability to bind to Gram-positive bacteria with a capture efficiency of 0.71 (±0.01) and 0.68 (±0.02) for *S. aureus* and *S. epidermidis*, respectively. A decreased capture efficiency was observed for Gram-negative bacteria by SiO_2_–MNPs and were 0.12 (±0.008) for *E. coli*-25922 and 0.05 (±0.008) for *E. coli*-DH5α. We propose the binding of SiO_2_–MNPs to bacteria occurs through hydrogen bonding by proteins on the bacteria cell membrane.^56^ Binding of silica nanoparticles to *S. aureus* and *E. coli* has been reported and molecular docking results showed that bacteria proteins attach to the surface of the silica *via* hydrogen bonding.^56^

The ability of Zn–DPA–SiO_2_–MNPs to bind both Gram-positive and Gram-negative bacteria with high affinity is remarkable. The binding can be explained by the presence of anionic phospholipids on the cell membranes of both Gram-positive and Gram-negative bacteria.^57^ The positively charged Zn–DPA groups on Zn–DPA–SiO_2_–MNPs are attracted to the negative groups on the bacteria cell membranes; indeed, the peptidoglycan layer in Gram-positive bacteria may present anionic groups, such as teichoic acids containing phosphate linked polymers.^32,58^ In Gram-negative bacteria, the lipopolysaccharides (LPS) with their anionic phosphate groups may interact with the positively charged Zn–DPA groups.^57–61^ Lee *et al.* using Zn–DPA polymer coated MNPs were able to bind *E. coli* with high capture efficiencies, like the Zn–DPA–SiO_2_–MNPs does.^28^ They did not report Gram-positive binding. In contrast, Zn–DPA–SiO_2_–MNPs shows the enhanced ability to bind quantitatively both Gram-negative and Gram-positive bacteria and do it without a polymer coating.

### 3.6 Bacteria binding in red blood cell suspension

**Fig. 9:**
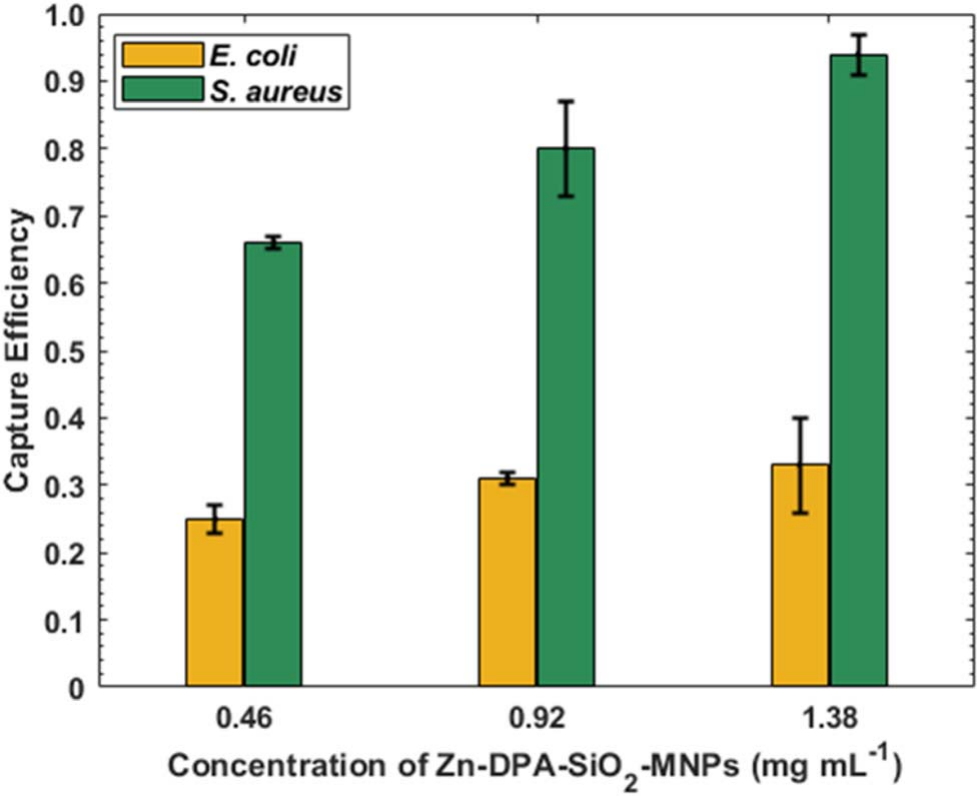
Zn–DPA–SiO_2_–MNPs capture efficiency with high concentrations of *S. aureus* (green) and *E. coli* (yellow) in RBC suspensions with 50% hematocrit. 0.46, 0.92, and 1.38 mg per mL MNP dispersions were used with 1.3 × 10^8^ CFU bacteria in RBC suspensions. Data are given as means with the error bars representing standard deviations (*n* = 3).

The feasibility of using Zn–DPA–SiO_2_–MNPs to remove bacteria from solutions of red blood cells was tested with Gram-positive and Gram-negative bacteria with a washed red blood cell (RBC) concentration 1.3 × 10^8^ CFU mL^−1^. Different concentrations of Zn–DPA–SiO_2_–MNPs were used to determine the capture efficiency. Diluted blood containing 50% hematocrit was used instead of PBS. When 0.46 mg mL^−1^ of Zn–DPA–SiO_2_–MNPs were added to solutions of bacteria and RBCs, the capture efficiency for *S. aureus* was 0.66, and for *E. coli* it was 0.25 (Fig. 9). An increase in the amount of Zn–DPA–SiO_2_–MNPs to 0.92 and 1.38 mg mL^−1^ resulted in 0.80 and 0.94 capture efficiency for *S. aureus*, and 0.31 and 0.33 for *E. coli*. The capture efficiency from different concentrations of Zn–DPA–SiO_2_–MNPs are not significantly different for *E. coli* (*p* = 0.57) and slightly different for *S. aureus* (*p* = 0.06). A larger amount (1.38 mg mL^−1^) of Zn–DPA–SiO_2_–MNPs was needed to obtain 0.94 removal of *S. aureus* unlike what was seen in PBS where 0.46 mg mL^−1^ of the Zn–DPA–SiO_2_–MNPs was sufficient to achieve quantitative capture efficiency. This inhibiting of binding of Zn–DPA–SiO_2_–MNPs to bacteria by RBCs is most likely due to Zn–DPA–SiO_2_–MNPs binding to RBCs. Initial studies of bacteria capture by Zn–DPA–SiO_2_–MNPs in whole blood were performed with Gram-positive bacteria (*S. aureus*) and no bacteria capture was observed under the initial conditions. The reason for this is not yet known, but we plan to carry out further capture studies to ascertain what component of the blood is inhibiting the binding of Zn–DPA–SiO_2_–MNPs to bacteria.

**Fig. 10:**
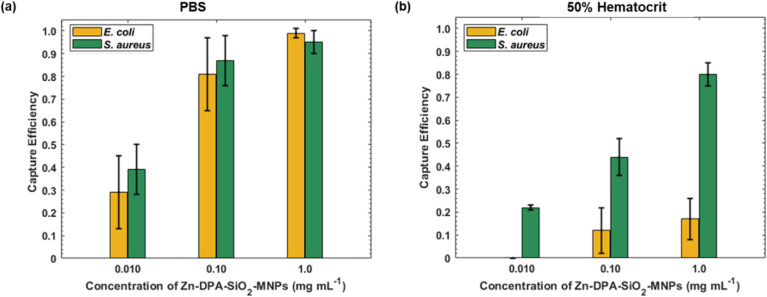
Zn–DPA–SiO_2_–MNPs capture efficiency with low concentrations of *S. aureus* and *E. coli*. 0.010, 0.10, and 1.0 mg per mL MNP dispersions were used with 1 × 10^3^ CFUs of bacteria in (a) PBS and (b) 50% red blood cell suspension. Data are given as means with error bars representing the standard deviations (*n* = 3–6).

Bacterial infections at their early stages have low bacteria concentrations. To discover the ability of Zn–DPA–SiO_2_–MNPs to bind low concentrations of bacteria we carried out binding studies with 1000 CFU mL^−1^ of bacteria in PBS and red blood cell suspensions. When 0.010 mg mL^−1^ of Zn–DPA–SiO_2_–MNPs were added to solutions of PBS and RBCs containing 1000 CFU mL^−1^ of *S. aureus*, the capture efficiencies were 0.39 in PBS and 0.22 in RBC (Fig. 10). The capture efficiency increased to 0.87 in PBS and to 0.44 in RBS when 0.10 mg mL^−1^ of Zn–DPA–SiO_2_–MNPs were used. It again increased and became 0.95 in PBS and 0.80 in RBC when 1.0 mg mL^−1^ of Zn–DPA–SiO_2_–MNPs were used. Low concentrations of the Zn–DPA–SiO_2_–MNPs were also used to bind low concentrations of *E. coli*. When 0.010 mg mL^−1^ of Zn–DPA–SiO_2_–MNPs were added to solutions of PBS and RBCs containing 1000 CFU mL^−1^ of *E. coli*, the capture efficiencies was 0.29 in PBS and no capture was observed in RBC (Fig. 10). The capture efficiencies increased to 0.78 in PBS, and to 0.12 in RBC when the concentration of the Zn–DPA–SiO_2_–MNPs was increased to 0.10 mg mL^−1^. The capture efficiencies further increased to 0.99 in PBS and 0.17 in RBCs when 1.0 mg mL^−1^ of Zn–DPA–SiO_2_–MNPs was used. The capture efficiency from the different concentrations of Zn–DPA–SiO_2_–MNPs shows a statistical significant difference in both PBS and RBC (*p* < 0.05). These results show that Zn–DPA–SiO_2_–MNPs can be employed for removal of bacteria (both low and high bacteria concentrations). Zn–DPA–SiO_2_–MNPs offers a solution for detecting and removing bacteria across a wide range of bacteria concentrations, making them potentially very useful in both clinical and industrial applications.

The successful capture of bacteria in PBS by Zn–DPA–SiO_2_–MNPs can be attributed to the cationic properties of Zn–DPA. The cationic Zn–DPA group will target the anionic phosphate groups present on lipoteichoic acids of the cell walls of Gram-positive bacteria. The anionic lipopolysaccharides (LPS) in Gram-negative bacteria are potential candidates for Zn–DPA binding. The reduced bacteria capture efficiencies of Gram-negative bacteria in RBC suspensions may be due to Zn–DPA–SiO_2_–MNPs binding to RBC membrane proteins such as glycoproteins. When RBCs and bacteria are both present, Zn–DPA–SiO_2_–MNPs binds to both, and thus isn't as efficient in binding bacteria. However, as the binding to Gram-positive bacteria in the presence of RBC is still efficient, this means Zn–DPA–SiO_2_–MNPs binds more strongly to Gram-positive bacteria. The reduced binding to Gram-negative bacteria could potentially also be due to physical shielding, electrostatic screening, and protein corona effects.^62^ Gram-negative bacteria are more greatly affected because their phosphate-rich sites are less exposed when compared to Gram-positive bacteria due to the difference in their cell membranes.^63^

The Zn–DPA–SiO_2_–MNPs capture of bacteria from PBS and RBC suspensions has implications across clinical, environmental, and industrial fields. From a clinical perspective, Zn–DPA–SiO_2_–MNPs could potentially be put into micro devices and used for early detection of bacteria. They could also potentially be used to purify biological fluids and reduce the risk of bloodstream infections, which would alleviate the need for antibiotics. From an environmental perspective, Zn–DPA–SiO_2_–MNPs could present a promising strategy for improving water quality. In PBS, Zn–DPA–SiO_2_–MNPs was very efficient in capturing bacteria and thus could potentially be used to purify drinking water and wastewater. In industrial settings, the selective removal of bacteria using Zn–DPA–SiO_2_–MNPs could potentially be applied to food items to reduce microbial contamination. Overall, the ability to bind and magnetically remove bacteria from suspensions positions Zn–DPA–SiO_2_–MNPs as a multipurpose material with potential to safeguard human health and protect the environment.

## 4 Conclusion

To improve bacterial removal from bodily fluids, MNPs have been synthesized, coated with silica and functionalized them with Zn–DPA. The new material, Zn–DPA–SiO_2_–MNPs bind well to a variety of both Gram-positive bacteria such as *S. aureus*, *S. epidermidis* and *P. aeruginosa*, and Gram-negative bacteria such as different strains of *E. coli*. Once bound to the MNPs, the bacteria are easily removed from PBS and RBC suspension with a magnet. The bacteria removal process only takes a few minutes, making it a faster method than the conventional method of collecting bacteria in body fluids.^5,64^ The bacteria binding may be useful for bacteria detection and removal from various fluids, which would make this new material useful for diagnostic tests.

## Author contributions

Tochukwu P. Okonkwo: synthesis of MNPs, synthesis of Zn–DPA–SVA, functionalization of MNPs, characterization of materials, data preparation, writing original draft, review and editing. Rajendra P. Gautam: magnetometry data collection and analysis. Jacob B. Limburg: synthesis of MNPs. Breckin L. Forstrom: bacterial binding assays. Bowen J. Houser: bacterial binding assays. Aaron Rappleyea: bacterial binding assays. Tyler P. Green: synthesis of MNP beads. Joseph P. Talley: synthesis of MNP beads. Alexander D. Daum: synthesis of Zn–DPA–SVA. Stacey J. Smith: XRD investigation and methodology. Karine Chesnel: conceptualization, methodology, writing, review, editing and funding acquisition. William G. Pitt: conceptualization, methodology, review, editing and funding acquisition. Roger G. Harrison: conceptualization, methodology, formal analysis, writing, review and editing, visualization, funding acquisition, and project administration.

## Conflicts of interest

All authors declare that they have no conflicts of interest with the research presented in this paper.

## Supplementary Material

RA-015-D5RA03701H-s001

## Data Availability

The data supporting this article have been included as part of the SI. Synthetic procedures for DPA–SVA; XPS data; UV/vis spectra; bead stability data; bacterial growth data; simplified capture efficiency for bacteria in PBS with SiO_2_–MNPs, DPA–SiO_2_–MNPs and Zn–DPA–SiO_2_–MNPs. See DOI: https://doi.org/10.1039/d5ra03701h.
