# Supplementary material for: Magnetic nanoparticles tethered with Zn–DPA for the removal of bacteria from red blood cell suspension
Source: RSC Adv. 2025 Sep 2;15(38):31402–15. doi: 10.1039/d5ra03701h (PMC12403053; doi:10.1039/d5ra03701h)
Supplement: RA-015-D5RA03701H-s001 [file RA-015-D5RA03701H-s001.pdf]

Supplementary Information (SI) for RSC Advances.
This journal is © The Royal Society of Chemistry 2025

SUPPLEMENTARY MATERIAL

Magnetic Nanoparticles Tethered with Zn-DPA for the Removal of Bacteria from Red Blood Cell Suspension

Tochukwu P. Okonkwo[1]

Rajendra P. Gautam[2]

Jacob B. Limburg[1]

Breckin L. Forstrom[3]

Bowen J. Houser[3]

Aaron Rappleyea[3]

Tyler P. Green[3],

Joseph Talley[3],

Alexander D. Daum[1]

Stacey J. Smith[1]

Karine Chesnel[2]

William G. Pitt[3]

Roger G. Harrison[1] *

*Corresponding author, roger_harrison@byu.edu

[1] Department of Chemistry and Biochemistry, Brigham Young University, Provo, UT USA

[2] Department of Physics and Astronomy, Brigham Young University, Provo, UT USA

[3] Department of Chemical Engineering, Brigham Young University, Provo, UT USA

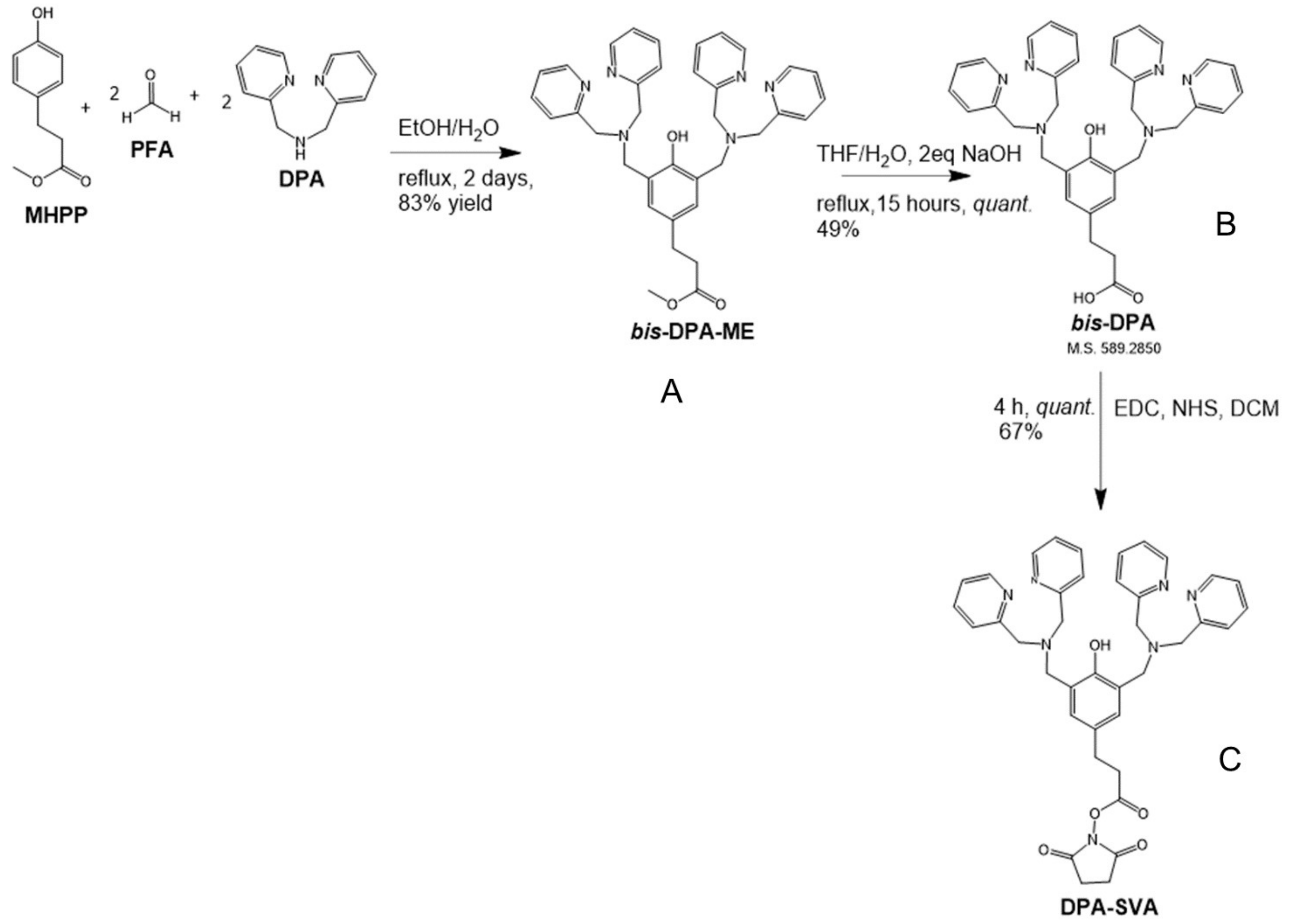

Figure S1: Synthetic Procedure for DPA-SVA.

S1(A) bis-DPA-ME

Paraformaldehyde (PFA, 0.5517 g, 18.4 mmol) and di(2-picolyl)amine (DPA, 2.7906 g, 14.0mmol) were added to a 250 mL round bottom flask and suspended in water (45 mL) and ethanol (15 mL). HCl (1.0 M, 1.4 mL) and methyl 3-(4-hydroxyphenyl)propanoate (MHPP, 1.1468 g, 6.4 mmol) were added to the mixture and refluxed for two days. The dark orange crude product obtained was cooled to room temperature, neutralized with saturated Na_2CO_3 and extracted with chloroform. The organic phase was dried with Na_2SO_4, the solvent was dried under reduced pressure, and purified via column chromatography (MeOH:CHCl3 5:95) to give 3-(3,5-bis((bis(pyridin-2-ylmethyl)amino)methyl)-4-hydroxyphenyl)propanoate (bis-DPA-ME) as a red-orange oil (3.1648 g, 5.3 mmol, 83% yield). MS: 601.2935. The synthesis of bis-DPA-Me was confirmed by 1H NMR spectroscopy (Figure S1).1H NMR (500 MHz, $CDCl_3$-d6): δ

[ppm]10.89 (bs, 1H), 8.52 (d, 4H), 7.60 (t, 4H), 7.50 (t, 4H), 7.12 (d, 4H), 7.03 (s, 2H), 3.86 (s, 8H), 3.78 (s, 4H),3.62 (s, 3H), 2.84 (t, 2H), 2.56 (t, 2H).

S1(B) bis-DPA

0.8158 g (1.4 mmol) of the bis-DPA-ME was added to 50 mL THF. 0.1068 g (2.7 mmol) NaOH was dissolved in 50 mL water. The solutions were combined and refluxed for 15 hours. 1 M HCl was added to neutralize the solution and bis-DPA was extracted with chloroform (0.3903 g, 0.7 mmol, 49% yield). MS: 587.2770. The synthesis of bis-DPA was confirmed by 1H NMR spectroscopy (Figure S1).1H NMR (500 MHz, $CDCl_3$-d6): δ 8.52 (d, 4H), 7.61 (t, 4H), 7.47 (t, 4H), 7.27 (d, 4H), 7.07 (s,2H), 3.85 (s, 8H), 3.70 (s, 4H), 2.82 (t, 2H), 2.57 (t, 2H).

S1(C) bis-DPA-SVA

0.3128 g (0.53 mmol) of bis-DPA, 1-ethyl-3-(3-dimethylaminopropyl)-carbodiiminehydrochloride (EDC, 0.2210 g, 1.42 mmol), and N-hydroxysuccinimide (NHS, 0.0701 g, 0.61 mmol) was added to 10 mL of dry DCM. The solution was stirred at room temperature for 4 hours, the solvent removed under reduced pressure and purified via column chromatography (MeOH: $CHCl_3$ 5:95) to obtain DPA-succinimidyl valerate (DPA-SVA, 0.2431 g, 0.35 mmol, 67%) as a red-orange oil. MS: 684.3024. The synthesis of bis-DPA-SVA was confirmed by 1H NMR spectroscopy (Figure S1).1H NMR (500 MHz, $CDCl_3$-d6): δ 8.52 (d, 4H), 7.62 (t, 4H), 7.49 (t, 4H), 7.24 (d, 4H), 7.07 (s,2H), 3.89 (s, 8H), 3.79 (s, 4H), 2.83 (t, 2H), 2.67 (s, 4H) 2.59 (t, 2H).

XPS SPECTRA

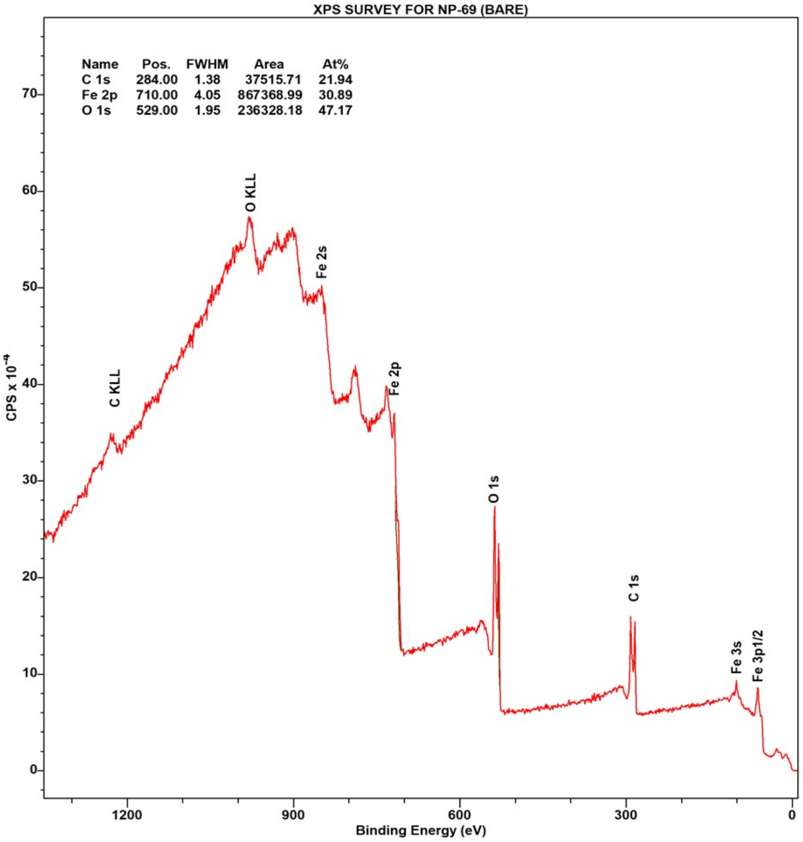

FigureS2: XPS spectra of the MNPs.

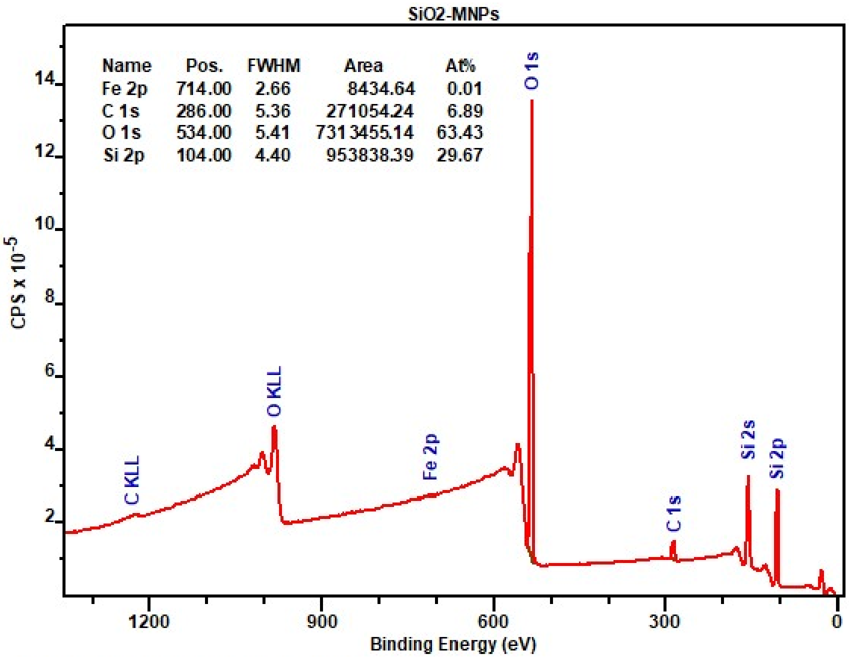

Figure S3: XPS spectra of SiO_2-MNPs.

Comment [BP]: What is this peak at ~160 eV that shows up in S3, S4, S5 and S6?

Comment [TO]: Its the Si 2s peak, I have included it in the plot

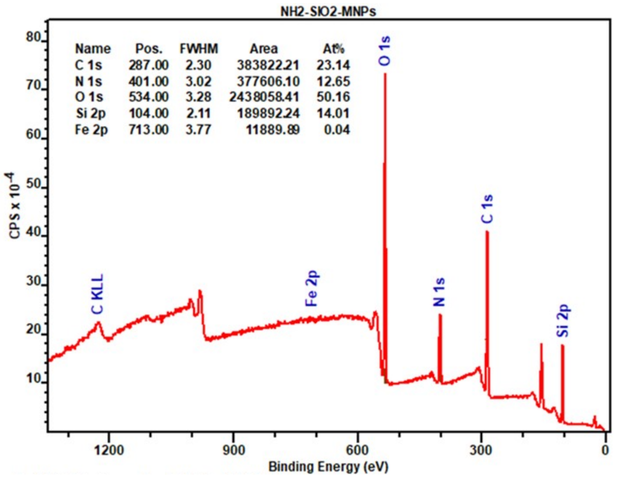

Figure S4: XPS spectra of NH_2-SiO_2-MNPs.

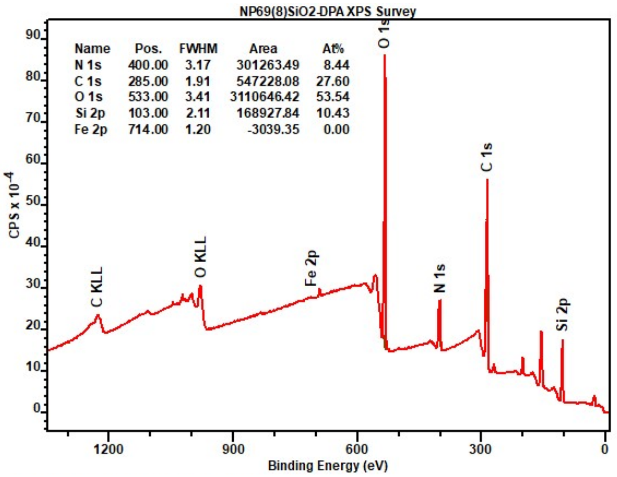

Figure S5: XPS spectra of DPA-SiO_2-MNPs.

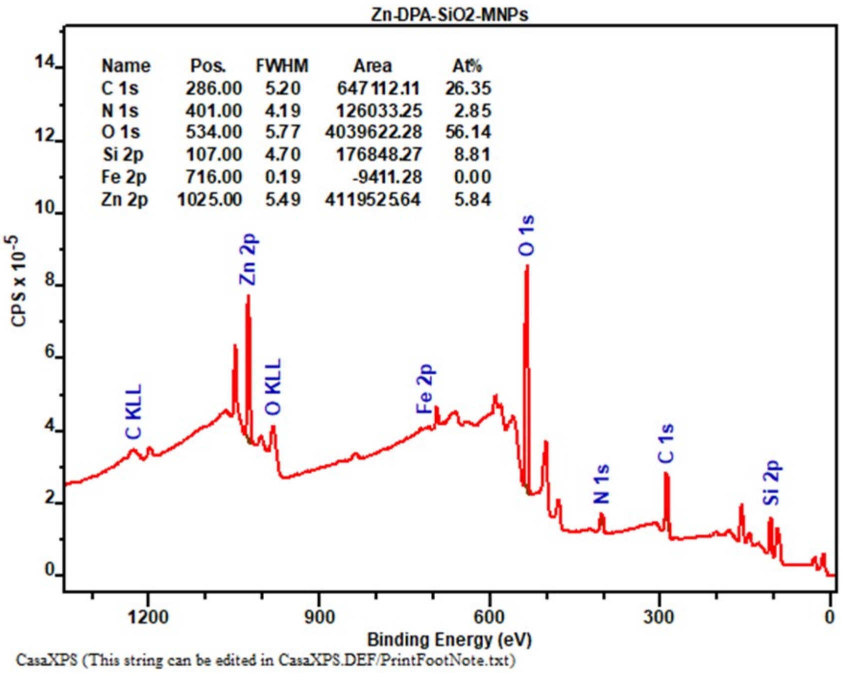

Figure S6: XPS spectra of Zn-DPA-SiO_2-MNPs.

Table S1: Bacterial growth media

Species	Plate media	Culture media
S. aureus	NA[1]	NB[1]
S. epidermidis	TSA[2]	TSB[2]
P. aeruginosa	LBA[3]	LBB[3]
E. coli DH5a	NA	NB
E. coli ATCC 225922	NA	NB

[1] Nutrient agar and broth

[2] Tryptic soy agar and broth

[3] Luria Bertani agar and broth

Table S2: Capture efficiency for bacteria (1.3×10^8 $CFUmL^{-1}$) in PBS with 2 mg/mL of SiO_2-MNPs, DPA-SiO_2-MNPs and Zn-DPA-SiO_2-MNPs with their standard deviation

Bacterial	SiO_2-MNPs	DPA-SiO_2-MNPs	Zn-DPA-SiO_2-MNPs
S. aureus	0.71 (± 0.016)	0.90 (± 0.003)	0.96 (± 0.01)
S. epidermidis	0. 68 (± 0.02)	0.79 (± 0.045)	0.98 (± 0.01)
P. aeruginosa	0.00	0.96 (± 0.015)	0.94 (± 0.01)
E. coli-DH5α	0.05 (± 0.008)	0.90 (± 0.04)	0.98 (± 0.012)
E. coli-25922	0.12 (± 0.008)	085 (± 0.025)	0.96 (± 0.007)

Table S3: Capture efficiency for bacteria (1.3×10^8 $CFUmL^{-1}$) in RBC with different concentrations Zn-DPA-SiO_2-MNPs with their standard deviation

Bacteria	Capture efficiencies for different masses of Zn-DPA-SiO_2-MNPs		
	0.46 mg/mL	0.92 mg/mL	1.38 mg/mL
S. aureus	0.66 (± 0.01)	0.80 (± 0.07)	0.94 (± 0.03)
E. coli -25922	0.25 (± 0.02)	0.31 (± 0.01)	0.33 (± 0.07)

Table S4: Capture efficiency for *S. aureus* and *E. coli* (1000 $CFUmL^{-1}$) in PBS with Zn-DPA-SiO_2-MNPs with their standard deviation

Bacteria in PBS	Capture efficiencies for different masses of Zn-DPA-SiO_2-MNPs		
	0.01 mg/mL	0.10 mg/mL	1.0 mg/mL
S. aureus	0.39 (± 0.11)	0.87 (± 0.11)	0.95 (± 0.05)
E. coli -25922	0.29 (± 0.16)	0.81 (± 0.16)	0.99 (± 0.02)

Table S5: Capture efficiency for *S. aureus* and *E. coli* (1000 $CFUmL^{-1}$) in RBCs with Zn-DPA-SiO_2-MNPs with their standard deviation

Bacteria in 50% Hematocrit	Capture efficiencies for different masses of Zn-DPA-SiO_2-MNPs		
	0.01 mg/mL	0.10 mg/mL	1.0 mg/mL
S. aureus	0.22 (± 0.0.01)	0.44 (± 0.08)	0.88 (± 0.05)
E. coli -25922	0.00	0.12 (± 0.10)	0.17 (± 0.09)

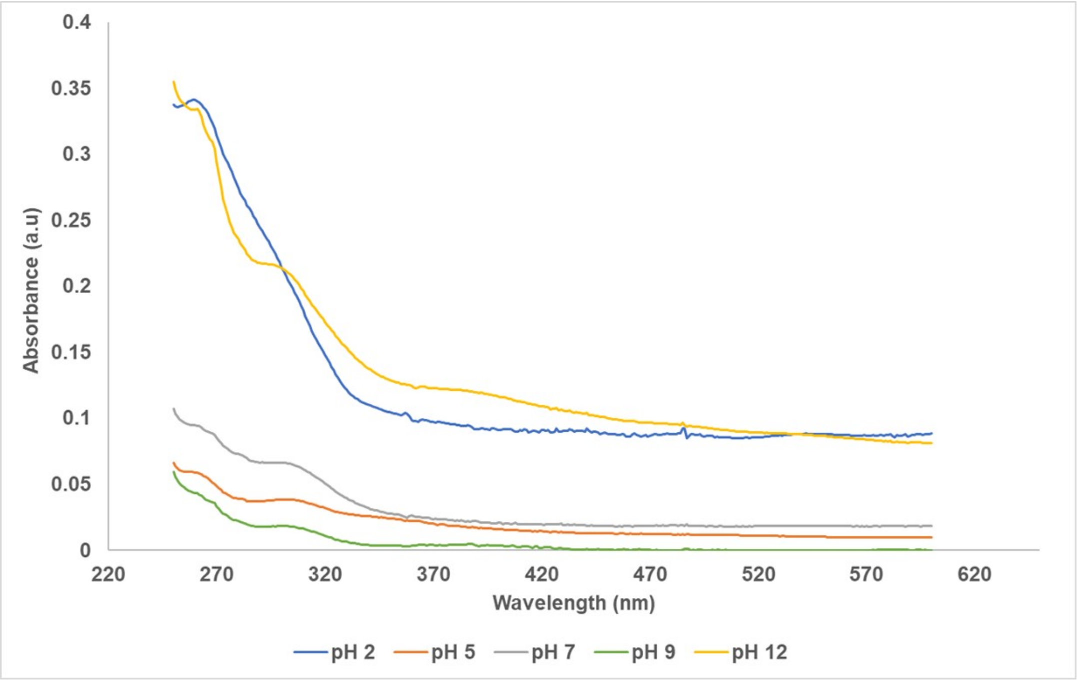

Figure S7: pH stability test of the Zn-DPA-MNPs using UV/Vis. Absorption spectra of supernatant at different pH values.

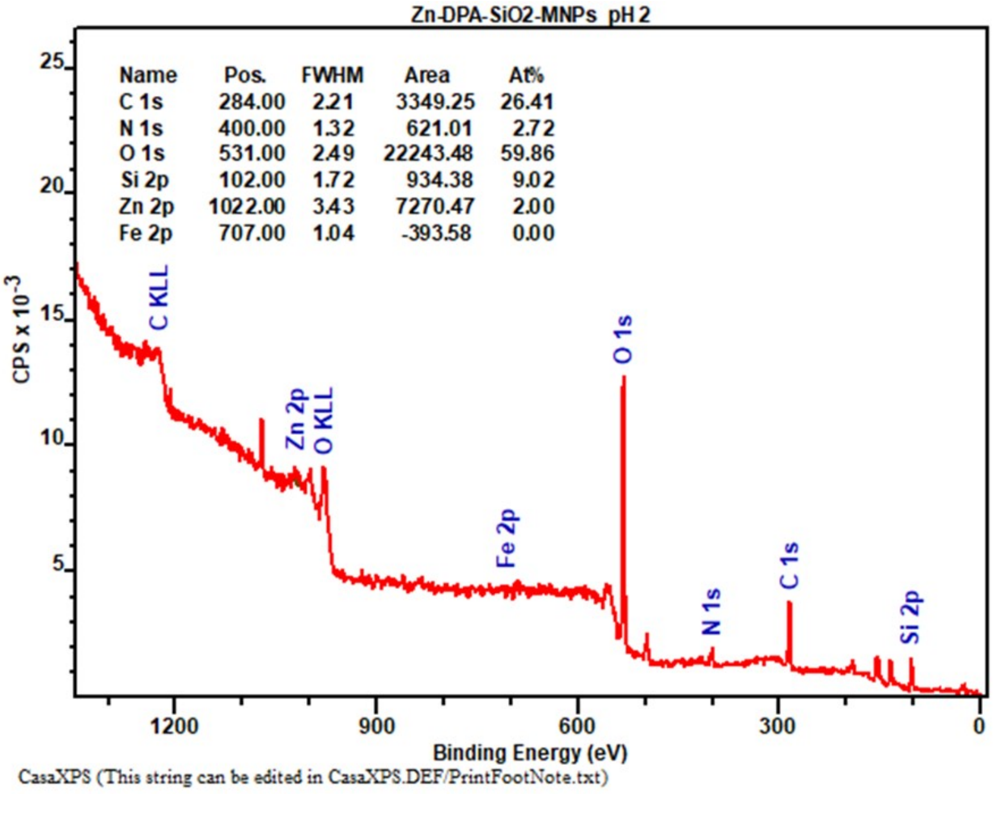

Figure S8: XPS spectra of Zn-DPA-SiO_2-MNPs from pH 2 solution.

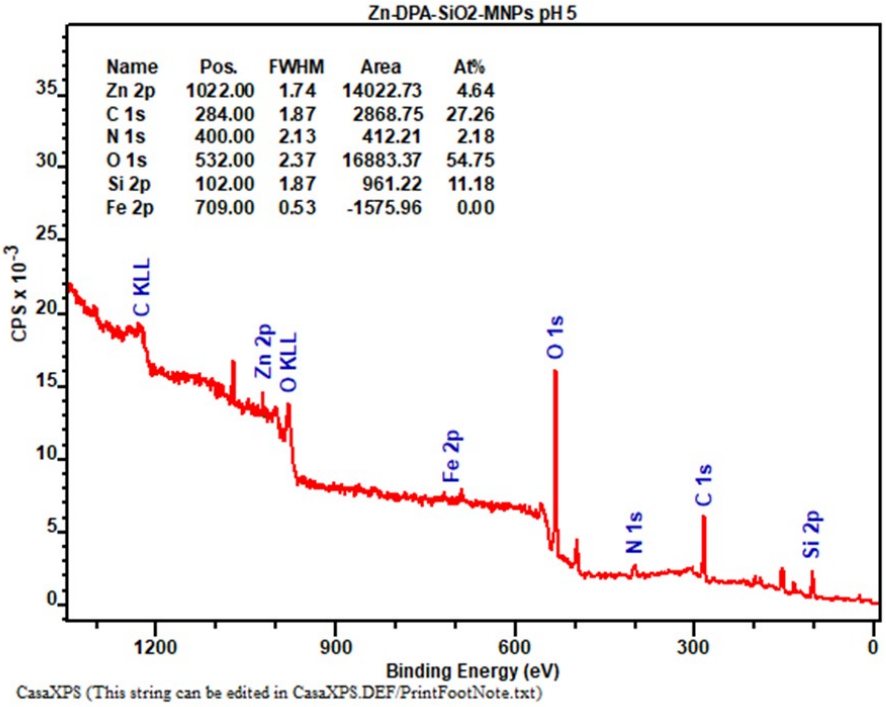

Figure S9: XPS spectra of Zn-DPA-SiO_2-MNPs from pH 5 solution.

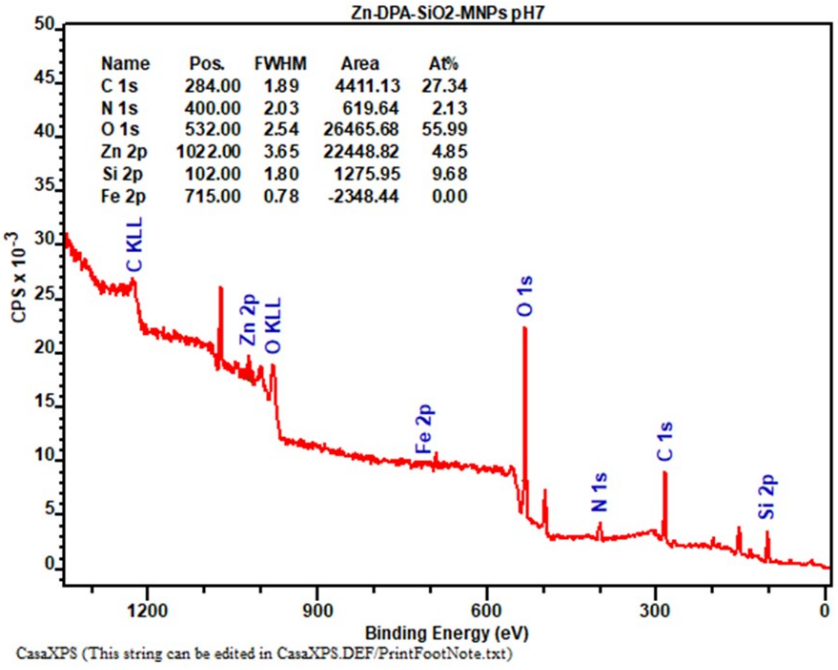

Figure S10: XPS spectra of Zn-DPA-SiO_2-MNPs from pH 7 solution.

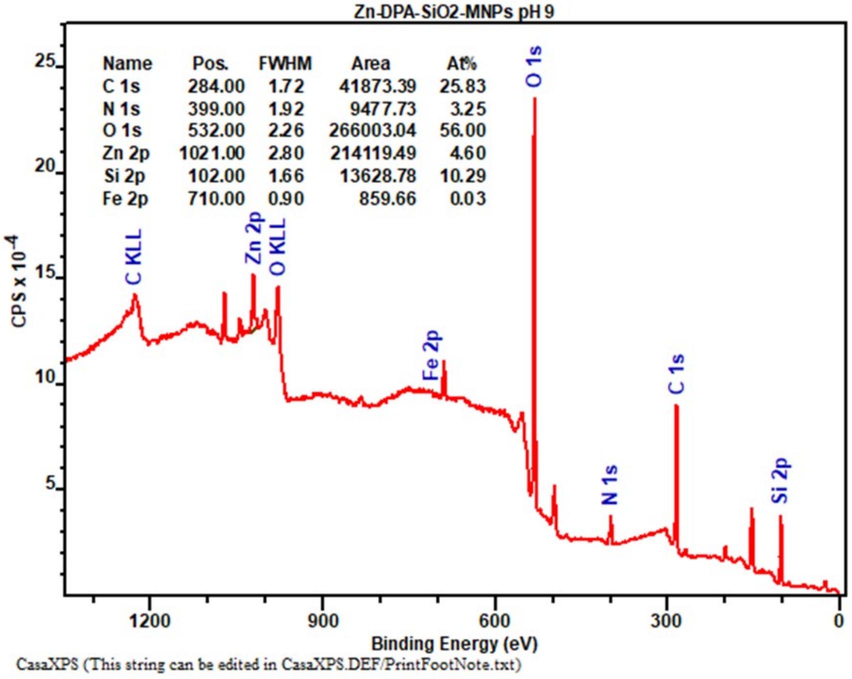

Figure S11: XPS spectra of Zn-DPA-SiO_2-MNPs from pH 9 solution.

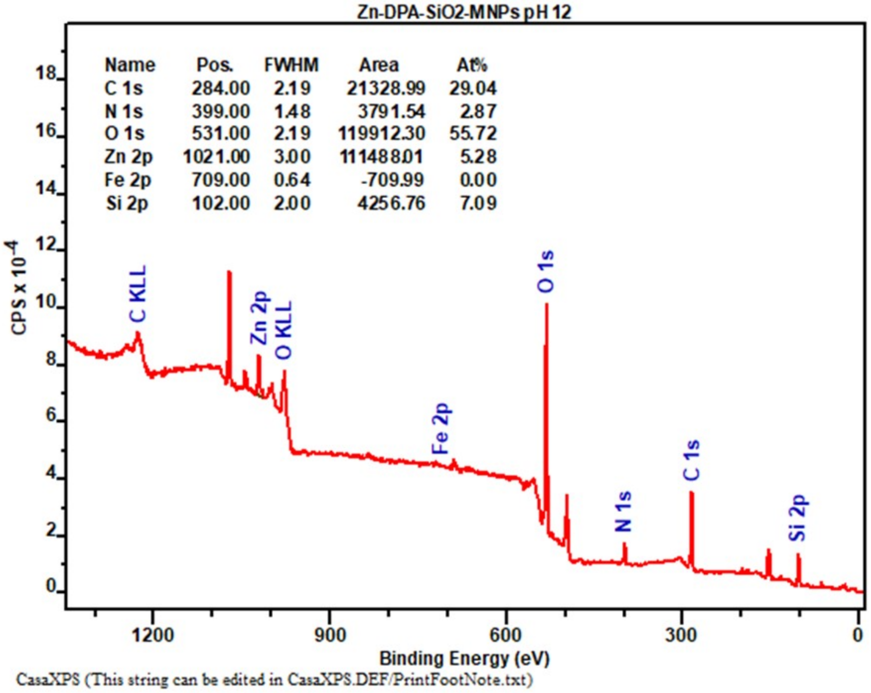

Figure S12: XPS spectra of Zn-DPA-SiO_2-MNPs from pH 12.
